# Crystallization of Cristobalite in Sodium Borosilicate Glass in the Presence of Cr_2_O_3_

**DOI:** 10.3390/ma16145016

**Published:** 2023-07-15

**Authors:** Marina Konon, Irina G. Polyakova, Anton S. Mazur, Artem S. Saratovskii, Dmitry P. Danilovich, Mikhail Alikin

**Affiliations:** 1 Grebenshchikov Institute of Silicate Chemistry, Russian Academy of Sciences, 199034 St. Petersburg, Russia; 2 Magnetic Resonance Research Centre, Saint Petersburg State University, 199034 St. Petersburg, Russia; 3 St. Petersburg State Technological Institute, Technical University, 190013 St. Petersburg, Russia

**Keywords:** borosilicate glass, phase separation, crystallization, Cr_2_O_3_, eskolaite, cristobalite, polytypism

## Abstract

Glass containing chromium is a promising material for use in various modern fields of application (laser technology, optoelectronic devices, and luminescent resources). Chromium oxides are well-known nucleating agents that can cause crystallization. One of the most commonly observed crystalline phases in silicate glasses is cristobalite, which lowers their mechanical strength, leading to the destruction of the material. The objective of this investigation was to study in detail the crystallization of cristobalite in sodium borosilicate glass in the presence of 2 mol% Cr_2_O_3_, depending on the thermal history of the glass. The glass was studied using XRD, SEM, EPR, FTIR-spectroscopy, XPS, and solid-state NMR. Eskolaite, α-Cr_2_O_3_, which had crystallized in this glass, stimulated the bulk crystallization of cristobalite at 550 °C after isothermally treating it for 72 h, due to the phase-separated structure of the glass with its interpenetrating phase morphology. Polytypism, resulting in the incorporation of alkalis into the cristobalite structure, was observed. Cr_2_O_3_ causes the catalytic crystallization of cristobalite at an extremely low temperature, which is at lower concentrations and temperatures than in glass containing Fe_2_O_3_ with a similar composition. The crystal growth rate and the incubation time for the crystallization of cristobalite were roughly estimated.

## 1. Introduction

In the past few decades, due to their exceptional electrical, optical, and magnetic properties, glasses doped with 3D-transition metal oxides (Ti, V, Cr, Mn, Fe, Co, Ni, and Cu) have attracted more and more interest from researchers, from a fundamental as well as a practical point of view. Transition metal ions (TMI) exist in glass in more than one valence and coordination state [1,2,3], which governs the unique physical properties of doped glasses, and, in turn, determines a wide range of their practical applications in areas such as fiber optic technologies, microelectronics [4,5,6,7], electro-optics [8], etc.

Of particular interest is the study of glasses doped with chromium oxides. Such glasses have interesting optical, electrical, and magnetic properties, due to the fact that chromium can exist in glasses in several oxidation states (+3, +4, +5, +6) [9,10]. Different valence states cause different coordinations of chromium ions with respect to oxygen, and, accordingly, their structural roles. The valence state of chromium ions determines the properties of such glasses, such as luminescence [11,12,13,14], semiconducting behavior [15], and magnetic properties [16]. These properties make it possible to consider glasses containing chromium as promising materials for use in various modern fields, such as laser technology, optoelectronic devices, and solar power engineering [9], in the tunable solid-state lasers that are used in telecommunication systems [17,18,19,20], electro-optical modulators, nonlinear optical and parametric converters, fiber optical amplifiers [21,22], for innovative luminescent resources [17], and in luminescent solar concentrators [23,24,25]. In addition, materials containing chromium ions have recently attracted interest as cathodes in storage batteries, due to their high energy density and high capacity [17,26]. Glasses modified with chromium oxides can also be used as effective protective screens against X-ray and gamma radiation [10,27]. The majority of these application fields require the materials for the intended use to possess a certain mechanical durability, to ensure the stable operation of the devices without the need to replace glass parts. However, chromium oxides are known to be nucleating agents that cause crystallization in glass [28], and this is not always a favorable occurrence.

For example, one of the crystalline phases that often forms in silicate glasses is cristobalite [29,30]. Cristobalite is one of the phases of silica and undergoes a reversible phase transition (α-β transformation) from a low-temperature tetragonal to a high-temperature cubic modification due to heating to temperatures of 120 to 270 °C, depending on the composition and amount of impurities in the silica; this change is accompanied by an abrupt increase in volume of ≈4–5% [31]. This volume change generates substantial mechanical stress that leads to crack formation during cooling and the subsequent destruction of glass samples, resulting in an inferior product that is unsuitable for practical applications that require mechanical strength [32,33]. This makes the crystallization of cristobalite an undesirable process in industrial glasses, especially considering how easily it forms in the presence of alkali oxides, which many glass materials contain [34]. For instance, alkali borosilicate glass is one of the most widely used glass families and has enormous utility. Among their many applications, these glasses are used in Pyrex (as laboratory glassware and household cooking utensils), glass seals, the Vycor process for making glasses with high silica content, large astronomical telescopes, automobile headlamps, and the immobilization of nuclear waste [35,36]. Due to the presence of two glass-formers, borosilicate glasses combine the advantages of the stability of silicate glass and the higher TMI solubility characteristic of borate glass, which makes them ideal host matrices for TMI such as chromium [15,37]. However, borosilicate glasses are also prone to cristobalite crystallization [29,38]. Its appearance was registered in sodium borosilicate (NBS) glasses containing iron oxides [38,39,40,41,42], alumina [43], and α-Si_3_N_4_ [44]. Our preliminary studies of chromium-containing NBS glass also showed the crystallization of cristobalite [45]. Cristobalite was suggested as forming in the boron-rich phase of a phase-separated glass during heat treatment in glasses containing iron and chromium [41,45], causing the glass to crack and break, thereby limiting the possibilities of potential applications. Therefore, the objective of this investigation was to study, in further detail, the crystallization of cristobalite in NBS glass in the presence of Cr_2_O_3_, depending on the thermal history of the glass.

## 2. Materials and Methods

### 2.1. Glass Synthesis

Glass with a composition of (mol %) 6Na_2_O·22B_2_O_3_·70SiO_2_·2Cr_2_O_3_ was synthesized using conventional melting techniques. The glass batch was a mixture of H_3_BO_3_ (p.a., Vekton, Russia), Na_2_CO_3_ (puriss., ECROS, Russia), Cr_2_O_3_ (p.a., Vekton, Russia), and SiO_2_ in the form of ground high-purity quartz glass (KV-glass, Russian state standard 15130-86, metal impurities ≤ 1 × 10^−2^ wt %, OH groups—(1.5–6) × 10^–2^ wt %). The glass was melted in a platinum crucible placed in an electric furnace with SiC heating elements, undergoing constant stirring of the melt with a platinum stirrer at 1450–1480 °C in air for 2 h. The melted glass was then poured onto a heated steel plate and transferred to an electric muffle furnace for annealing (temperature 510 °C, duration—5 min). After annealing, all glasses were additionally heat-treated in a muffle furnace at 550 °C for 24–96 h and at 700 °C for 2 h to promote phase separation and crystallization. A separate quenching experiment was carried out, wherein the glass melt was poured into an ice-cold water bath.

### 2.2. X-ray Powder Diffractometry (XRD)

The diffraction patterns for the investigated glasses were obtained using the Rigaku SmartLab 3 multifunctional powder diffractometer (Rigaku Corporation, Tokyo, Japan), CuK_α_ radiation. Crystalline compounds were identified according to powder diffraction files, using the PDF-2 database.

### 2.3. Scanning Electron Microscopy (SEM)

Scanning electron microscopy (SEM) (TESCAN, VEGA 3SBH, Tescanbrno, s.r.o., Brno, Czech Republic) was used to study the microstructure of the glass samples. A freshly fractured surface of a glass sample was preliminarily etched in a 5% aqueous HF solution for 5 s. Then, a thin layer of carbon was deposited on the etched surface of the sample, using magnetron sputtering to increase the efficiency of charge-draining. The surface morphology was analyzed using a secondary electron detector, then the distribution of components was analyzed using a backscattered electron detector in the contrast mode according to the average atomic number. The accelerating voltage was 30 kV.

### 2.4. Electron Paramagnetic Resonance (EPR)

Electron paramagnetic resonance (EPR) spectra were recorded for powdered glass samples (passed through 0.14–0.20 mm sieves) using the Bruker ELEXSYS E580 EPR spectrometer (Bruker Optik GmbH, Karlsruhe, Germany) operating in the X-band (9.46 GHz) with a modulation frequency of 100 kHz at 100 K. The microwave power used was 1.5 mW.

### 2.5. Fourier-Transform Infrared (FTIR) Spectroscopy

The FTIR spectra were recorded with a Shimadzu IRTracer-100 Fourier transform infrared spectrometer (Shimadzu, Kyoto, Japan), using samples in the form of tablets that were 10 mm in diameter, pressed from a mixture of the sample and KBr; the ratio of the sample to KBr was 1:100. All spectra were recorded on 32 scans in the range of 4000 to 350 cm^−1^, with a resolution of 2 cm^−1^, and the scans were taken at room temperature (20 °C).

### 2.6. X-ray Photoelectron Spectroscopy (XPS)

The XPS measurements were performed at the “Physical Methods of Surface Investigation” Resource Center of St. Petersburg State University, using a photoelectron spectrometer, the Escalab 250Xi, with AlKα radiation (photon energy 1486.6 eV). Spectra were recorded in the constant pass energy mode at 50 eV for the survey spectrum and at 20 eV for the element core level spectrum, using an XPS spot size of 650 μm. The total energy resolution of the experiment was about 0.3 eV. The investigations were carried out at room temperature in an ultrahigh vacuum, of the order of 1 × 10^−9^ mbar. An ion-electronic charge compensation system was used to neutralize the sample charge.

### 2.7. Solid-State Nuclear Magnetic Resonance (NMR)

The NMR analyses were conducted using the Brucker Avance III 400 WB (9.4 T) spectrometer. The crushed glass samples (the same as those used for the EPR experiments) were placed in a zirconium oxide rotor with an external radius of 4 mm. The rotor was spinning at a frequency of 12.5 kHz at a magical angle to a constant magnetic field. Studies were carried out on the ^11^B, ^23^Na, and ^29^Si nuclei. H_3_BO_3_, NaCl solution in H_2_O, and (CH_3_)_4_Si were used as the external reference, respectively. For all nuclei, when registering the spectra, a one-pulse sequence was used with pulse lengths equal to 3.3 μs, 1.2 μs, and 2.5 μs, along with relaxation delays of 0.5 s, 0.5 s, and 30 s for the above nuclei, respectively.

## 3. Results

### 3.1. XRD Results

**Figure 1:**
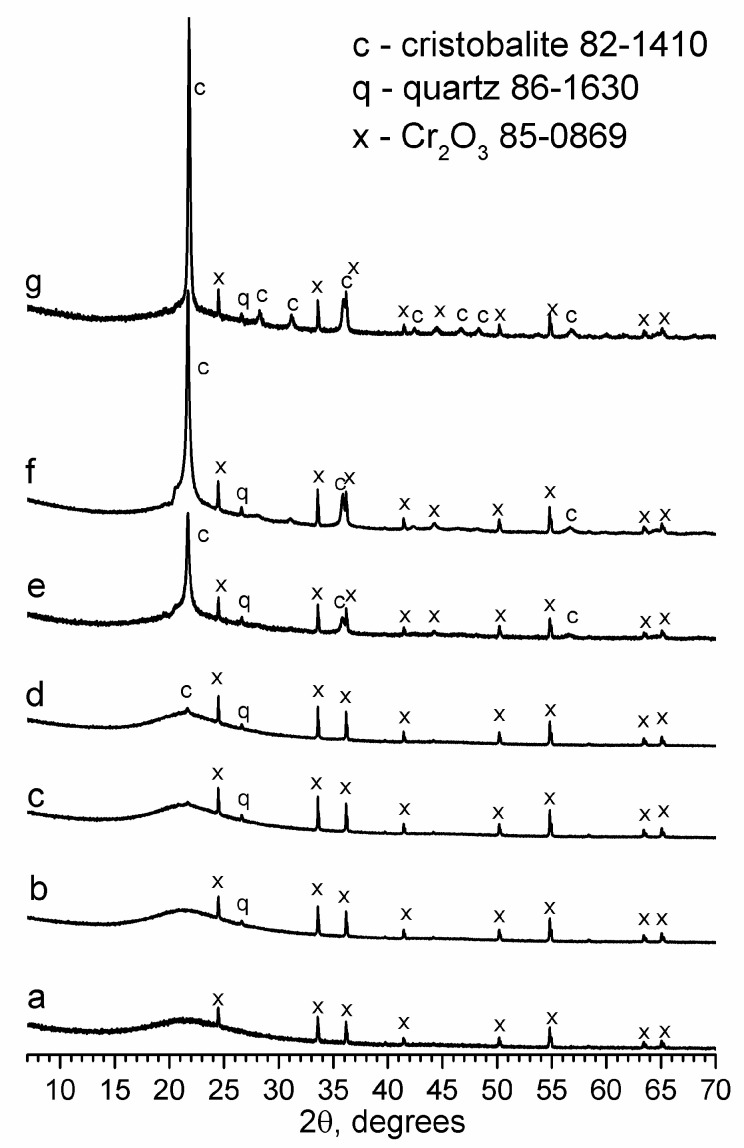
XRD patterns for the investigated glass samples with different thermal histories: quenched (**a**) and annealed (**b**); heat-treated at 550 °C for 24 h (**c**), 48 h (**d**), 72 h (**e**), and 96 h (**f**); heat-treated at 700 °C for 2 h (**g**).

According to the XRD results (Figure 1), the only chromium-containing crystalline phase is α-Cr_2_O_3_ (85-0869, eskolaite). It appeared in the samples with all the studied thermal histories, including the quenched sample (Figure 1a), indicating a very rapid crystallization rate. After annealing and heat treatment at 550 °C for 24 h, the intensity of the eskolaite peaks grew slightly; moreover, starting from 2θ = 37°, the K_α1_–K_α2_ doublet of the copper anode of the X-ray tube was resolved, indicating the crystallization of well-formed, defect-free, and relatively large chromium oxide crystals. For annealed glass and all the heat-treated samples, traces of quartz (86-1630) were also detected, which had been introduced via the raw materials.

The formation of cristobalite started when the heat treatment duration reached 48 h, when a small main peak of cristobalite (82-1410) phase at 2θ = 21.9° appeared on the XRD pattern. With the increase in the duration of thermal treatment to 72 h and, then, to 96 h, the intensity of the main peak increased drastically, and other weak peaks of cristobalite appeared. The main peak intensity for the sample heated to 550 °C for 96 h was two times greater than the sample heated to 550 °C for 72 h. The crystallization of cristobalite was even more intense at higher temperatures (700 °C).

A broad “hump” was also observed at about 2θ = 20.7°, next to the main cristobalite peak for the samples that were heat-treated at 550 °C, which was absent in the XRD pattern for the glass treated at 700 °C.

### 3.2. SEM Results

**Figure 2:**
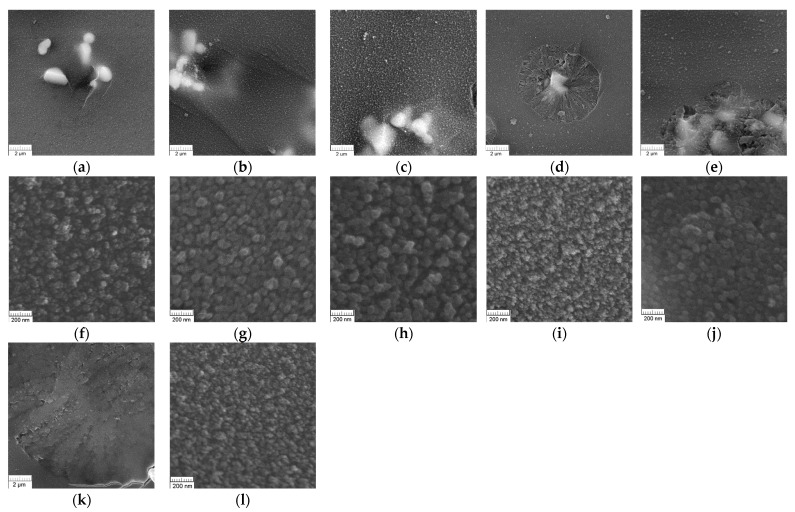
SEM images for the investigated glass samples with different thermal histories and magnification. The 20 k magnification images are (**a**–**e**,**l**), while the 100 k magnification images are (**f**–**k**,**m**). Annealed glass: (**a**,**f**) glass samples were heat-treated at 550 °C for 24 h (**b**,**g**), 48 h (**c**,**h**), 72 h (**d**,**i**), and 96 h (**e**,**j**). Images (**k**,**l**) show the glass samples heat-treated at 700 °C for 2 h.

The SEM images show that heat treatment of the investigated glass led to the formation of an interconnected phase-separated structure (Figure 2f–j,l) for all heat treatment conditions. The diameter of the connective boron-rich phase channels changed insignificantly with the increasing duration of the heat treatment (at 550 °C) and was recorded at around 40 nm, on average.

In addition to the phase-separated structure, all the studied glass samples contained crystalline inclusions (Figure 2a–e,k). When photographed in contrast mode according to average atomic number, these inclusions had the lightest shade detected, which indicated that they contained chromium ions. It was found that with an increase in the duration of heat treatment at 550 °C (Figure 2), the light-colored inclusions increased in size (from 0.8–1.2 µm, in glass heat-treated for 24 h, to 1.2–1.8 µm in a sample heat-treated for 72 h). After an isothermal exposure of 72 h, a new type of crystalline inclusions appeared in the form of spherulites with a diameter of ~7 μm; these can be attributed to cristobalite [29], a finding that is consistent with the XRD results. Light-colored inclusions were observed for samples treated for 72 and 96 h (Figure 2d,e), appearing in the centers of the spherulite formations. With a further increase in the duration of heat treatment to 96 h, the spherulite inclusions became coarser and their clusters appeared at sizes of about 15 μm. After heat-treating the glass at 700 °C, the micrographs also showed individual spherulitic inclusions of 12–13 µm in diameter, along with clusters of 22–24 µm in size. At the same time, no light-colored inclusions were seen in the centers of the spherulites during this heat treatment.

### 3.3. EPR Results

**Figure 3:**
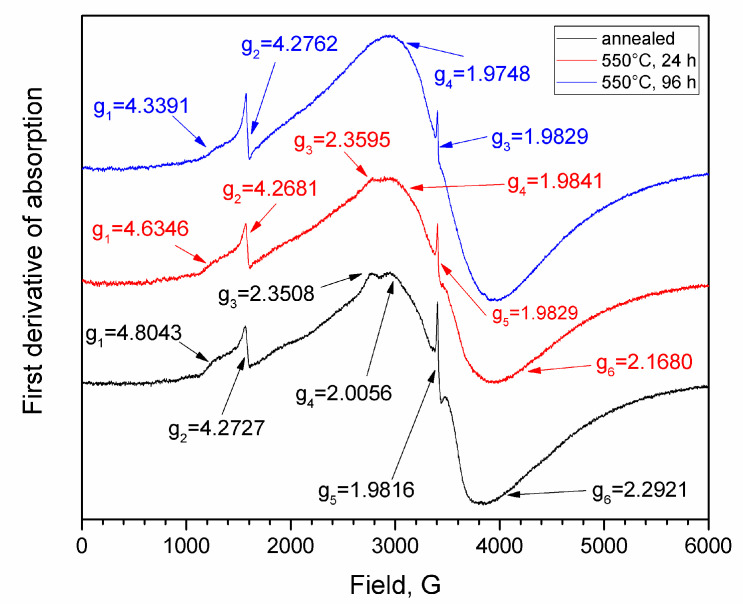
EPR spectra for the investigated glass samples with different thermal histories.

The EPR spectra of all the investigated samples had a complex shape and contained several resonance lines (Figure 3): a narrow asymmetric resonance with a *g*-factor of ~1.98, along with several wide resonance bands in the regions of *g* = 4.27, *g* = 2.00–2.35, and *g* = 4.6–4.8. The signal at *g* = 4.27 was associated with the presence of contaminants in the glass batch, namely, unintentional Fe^3+^ impurities that are often present in glass [46,47]. The resonance line with *g* = ~1.98 is characteristic of Cr^3+^ ions that are strongly coupled by antiferromagnetic exchange interactions and magnetic dipolar interactions or large clusters of Cr^3+^ [3,48]. Sometimes, this signal is also ascribed to contributions from both Cr^3+^ and Cr^5+^ ions [3,49]. The *g* value of 2.00 is attributed to the isolated Cr^3+^ centers at the axially distorted octahedral sites [46]. EPR lines with g-factors of around 4.6–4.8 can be assigned to the isolated Cr^3+^ centers in the strongly distorted octahedral sites of the glass network [3,46,50].

### 3.4. FTIR Spectroscopy Results

The FTIR results for the investigated glasses were compared with the FTIR spectra for the NBS glass without chromium additive, with a composition of 6Na_2_O·24B_2_O_3_·70SiO_2_ (designation—6/70) that was synthesized according to the procedure described in [51]. Samples of pure cristobalite and tridymite were also used as references; these were obtained from the Institute of Quartz Glass and were synthesized by G.A. Pavlova. The FTIR spectra for these samples and the 6/70 glass had not been obtained previously; they were recorded for the first time during the course of this work. Their characteristic bands are presented in Table 1.

**Figure 4:**
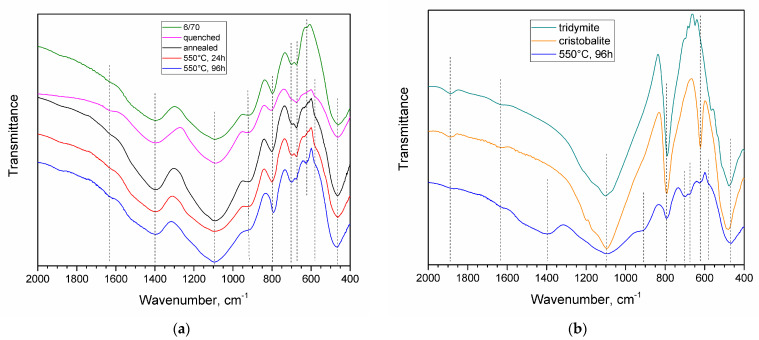
FTIR spectra for the investigated glass samples with different thermal histories, compared to a glass without Cr_2_O_3_ and with the composition (mol. % as synthesized) of 6Na_2_O·24B_2_O_3_·70SiO_2_ (6/70) (**a**). FTIR spectra for the investigated glass, heat-treated at 550 °C for 96 h, compared to cristobalite and tridymite (**b**).

The FTIR spectra of the investigated glasses (Figure 4a) showed transmission bands that are characteristic of borosilicate glasses (Table 1). Bands were detected that are typical for vibrations in the silicon-oxygen groups, with minimums at 461–464, 650–550, 633, 800, and 1091 cm^−1^. With an increase in the duration of heat treatment at 550 °C, the bands at 633 and 804 cm^–1^, which are characteristic of the symmetric stretching vibrations of Si−O−Si, shifted to lower wave numbers, then, at 96 h, they corresponded to the characteristic bands of cristobalite at 620 and 793 cm^–1^, respectively (Figure 4b, Table 1), correlating with the XRD results. All the chromium-containing samples exhibited a band at 582 m^−1^, which is typical for O-Cr-O vibrations in Cr_2_O_3_. 

There were also transmission bands that are characteristic of the vibrations of B-O-Si bonds (675, 911 cm^−1^) and the vibrations of B-O-B bonds in the trigonal groups of BO_3_ (675, 700, and 1400 cm^−1^). The band of BO_4_ tetrahedral group vibrations usually appears in the 1100–900 cm^−1^ region of the spectrum, but in borosilicate glasses, it is overlapped by the band of stretching vibrations of SiO_4_; therefore, it is impossible to assess the presence of four-coordinated boron in these glasses using only the IR spectroscopy data [52].

**Table 1:** FTIR band positions and respective assignments for the studied samples.

Band Position	Glasses’ Designations							Band Assignment	Ref.
	Quenched	Annealed	550 °C, 24 h	550 °C, 96 h	6/70	Cristobalite	Tridymite		
550–400	461	464	463	464	459	480	476	δSiO_4_ bending vibrations in the silicon-oxygen groups	[53,54,55]
650–550							565		
	582	582	582	582				Cr_2_O_3_ typical of O-Cr-O vibrations	[56,57]
		633	632	626	625	621	648	Si−O−Si, symmetrical stretching vibrations of the individual SiO_4_ tetrahedra of the polymerized structure of framework silicates such as α-cristobalite	[58]
800–650	673	675	675	676	675		679	B-O-Si stretching; δBO_3_ bending vibrations of bridging oxygen between trigonal BO_3_ groups	[15,53,59,60]
	700	699	699	699	700		696	δBO_3_; bending vibrations of B-O-B linkage in borate network	[53,61,62,63,64]
	804	800	800	793	799	793	789	Bending motions of the Si-O-Si bridges; α-cristobalite	[53,58,65]
1200–800	922	922	924	910	924			B-O-Si stretching mode ν (B–O–Si)	[66]
	1090	1092	1093	1095	1092	1096	1103	antisymmetric stretching vibrations of the O–Si–O bonds in SiO_4_ tetrahedral units for tetrahedra with three bridging oxygen ions (*Q*^3^)	[53,55]
						1198			
1500–1200	1396	1397	1399	1399	1396			B-O stretching vibrations involving B-O-B linkages associated with triangularly and tetrahedrally coordinated boron atoms; bending and stretching modes of the B–O bonds in the BO_3_ triangles	[62,64]
2000–1500	1630		1630	1623		1636	1636	OH symmetrical bending deformation	[67]

### 3.5. XPS Results

**Figure 5:**
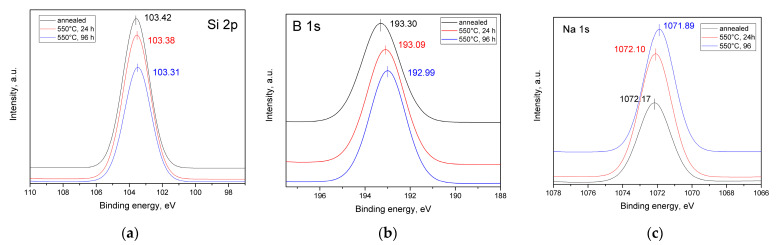
Si2p (**a**), B1s (**b**), and Na1s (**c**) photoelectron spectra for the annealed glass and those samples that were heat-treated at 550 °C for 24 and 96 h.

All the B1s, Si2p, and Na1s signals were expressed by one symmetric function (Figure 5) and shifted slightly toward the lower energy side with the increasing duration of the heat treatment. Only the B1s spectra shifted more significantly than the others; the difference between the binding energy peak for the annealed glass and the sample heat treated for 96 h (maximum duration) was 0.31 eV, which exceeded the total energy resolution. This might suggest a change in the bond configurations of the next-nearest boron neighbors [68]. 

**Figure 6:**
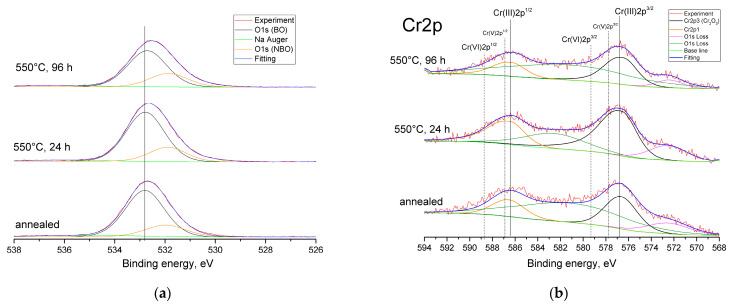
O1s (**a**) and Cr2p (**b**) photoelectron spectra for the annealed glass and those samples that were heat-treated at 550 °C for 24 and 96 h.

Oxide ions in the NBS system exhibit different types of chemical bonds; therefore, it is possible to analyze the bonding states of oxide ions by deconvoluting the O1s spectrum. The NBS glass should have various chemical bonds, such as Si-O-Si, Si-O-B_n_, B_n_-O-B_n_ (where *n* = 3 or 4 represents the coordination number of oxides around a boron atom) for the bridging oxygen (BO) component, and two types, Si-O^-^Na^+^ and B_3_-O^-^Na^+^, for the non-bridging oxygen (NBO) component [69]. The O1s spectra were fitted with BO and NBO components (Figure 6a), where the binding energy values of the BO component were around 532.7–532.8 eV (Table 2), and the binding energy values of the NBO component were higher than 531 eV (531.86–531.98 eV). 

There was no change in these values according to the duration of the heat treatment. In general, if the binding energy of O1s shifts toward lower values, this is interpreted as an increase in the electronic density of the oxide ions [69]. Electronic density can increase when the amount of four-coordinated boron increases; therefore, the large drop in the binding energy of O1s can be attributed to the formation of BO_4_ groups. The lesser drop seen in O1s can be attributed to the conversion of BO_4_ to BO_3_ groups with NBOs. However, in the investigated samples, the binding energy values only changed slightly, which could indicate a lack of the formation of BO_4_ groups with the increasing duration of heat treatment. The binding energy values for the NBO component were higher than 531 eV, which, according to the authors of [69], allowed them to be assigned exclusively to the NBO attached to the silica network. The binding energy of the Si2p peak for the sample that was heat-treated for 96 h was closest to the binding energy seen in α-cristobalite (103.3 eV) [70].

XPS was also used to analyze the valence state of the Cr ions (Figure 6b). The binding energies corresponding to Cr(VI) 2p1/2 and Cr(VI) 2p3/2 are usually located at 588.7 and 579.3 eV, respectively. The binding energies for Cr(III) 2p1/2 were −586.4 eV, and were 576.5 eV for Cr(III) 2p3/2 [70,71,72]. The standard binding energy for a Cr(V) compound (Ca_3_(CrO_4_)_2_) can be found in the NIST XPS database, while the binding energies for 2p1/2 and 2p3/2 are 577.8 and 586.9 eV, respectively [72]. For all the investigated glass samples, only the characteristic peaks of Cr(III) were detected. Furthermore, the binding energy of the O1s spectrum was also closer to that of Cr_2_O_3_ than any other chromium oxide [70]. 

### 3.6. NMR Results

The shape of the ^23^Na NMR spectra was heterogeneously broadened (not shown), which may be associated with the presence of a quadrupole interaction. In general, the maximum of the line shifted toward the positive values of the chemical shift, while the width of the resonant line decreased with an increase in the duration of heat treatment.

**Figure 7:**
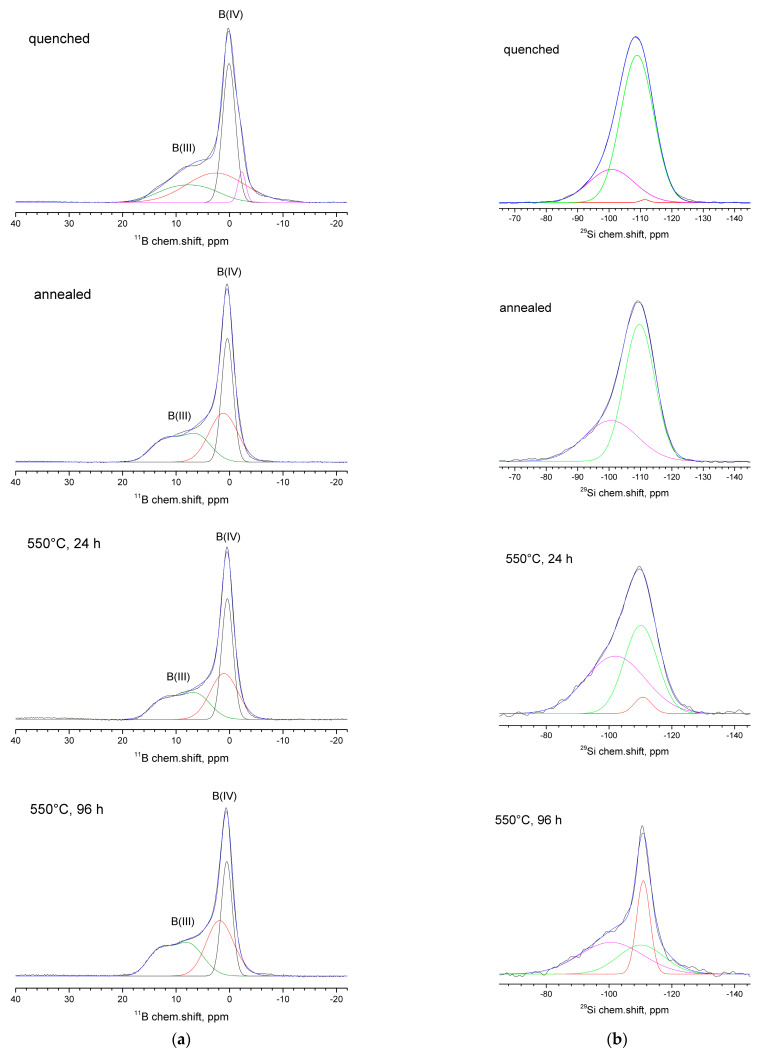
^11^B NMR spectra (**a**) and ^29^Si NMR spectra (**b**) for the investigated glasses with different thermal histories. For ^11^B spectra the colors are attributed in the following way: Black—BO_4_(3Si, 1B) units, blue—fitting for the experimental data, red—BO_3 non-ring_ units, magenta—BO_4_(4Si) units, green—BO_3 ring_ units. For ^29^Si spectra: blue—fitting for the experimental data, green—Q^4^(3Si, 1BO_3_) units, magenta—Q^4^ (3Si,1BO_4_) untis, red—Q^4^(4Si) units.

All the ^11^B NMR spectra of the studied samples have an intense peak at about 0 ppm and a broad peak at higher chemical shifts (Figure 7). For the quenched sample, a slight broadening of the narrow peak toward the negative values of the chemical shift was observed. Decomposing the spectra into their expected spectral components showed that all the studied samples contain three- and four-coordinated boron structural units. A narrow peak near the 0 ppm point corresponded to the BO_4_(3Si,1B) structural units (where a boron atom replaces a silicon atom) [73]. For the quenched sample, a small contribution from the BO_4_ (4Si) structural units (where the tetrahedral boron atom is surrounded only by silicon atoms) was also observed at about −2 or −3 ppm [74]. The broadest peak seen at the highest chemical shift values corresponded to three coordinated boron atoms and can be decomposed into two components. One of these components has a complex shape, with two well-defined maxima, and is located in the region of the spectrum with large chemical shifts. This component can be associated with boron that is surrounded by bridging bonds with the boron atoms, generally known as boroxol rings (BO_3 ring_), which could include other boron entities involving BO_3_–O–BO_4_ bonds [73,74]. The second component was visually indistinguishable in the spectrum and was revealed only as a result of the decomposition of the spectrum into its components. It has a broadened shape compared to BO_4_ and is located between the BO_3ring_ and BO_4_(3Si,1B) lines. This component can be attributed to boron that is distributed in silicate units (generally referred to as BO_3 non-ring_) [73,74]. Analyzing the areas of the corresponding spectral components (percentage of structural units) for all the samples being studied showed that with an increase in the duration of the heat treatment, a decrease in the BO_4_ structural units occurred, due to an increase in three coordinated structural units (Table 3). In this case, for all the treated samples, the ratio of BO_3 ring_ and BO_3 non-ring_ atoms did not change, practically speaking, although a reduced content of BO_3 ring_ atoms was observed for the quenched sample.

The ^29^Si NMR spectra of the studied samples were located in the range of chemical shifts characteristic of four coordinated silicon atoms (Figure 7b). The spectra were broadened to the positive values of the chemical shift. The decomposition of the spectra into the Gaussian peaks showed the presence of three components (except in the case of the annealed sample) at about −100.6 ppm, −110.2 ppm, and −110.9 ppm. The third component at −110.9 ppm was significantly narrower than the first two, indicating that the local environment of these silicon atoms was more symmetrical. The contribution at −100.6 ppm may have corresponded to the Q^4^ structural units of the Q^4^ (3Si and 1BO_4_) type. The component at −110.2 ppm could be attributed to the Q^4^ structural units of the Q^4^(3Si and 1BO_3_) type. The component at −110.9 ppm could be attributed to the structural Q^4^ units of the Q^4^ (4Si) type [73]. As the duration of the heat treatment increased, the number of structural units of type Q^4^ (3Si, 1BO_3_) was reduced, first, due to an increase in the structural units of Q^4^ (3Si, 1BO_4_), and second, due to a marked increase in the number of structural units of Q^4^ (4Si) (Table 3). This increase in the Q^4^ (4Si) structural units may be associated with the formation of the nanosized phase of cristobalite in the glass matrix [75].

## 4. Discussion

Cristobalite and tridymite are two of the most common tetrahedral silicas. Although quartz is not only the most well-known modification of silica but is also the most common mineral on Earth, from the point of view of the phase diagram for normal pressure, the main phase of silica is cristobalite. Cristobalite is distinguished by its high purity and low solubility of other chemical elements (mainly alkali). Tridymite cannot be considered a pure silica phase because it needs impurities or water to stabilize it [31]. However, cristobalite is known to contain tridymite-type stacking faults in the presence of impurities, mainly alkalis [34], where the intergrowing of cristobalite and tridymite structures is observed. Depending on the close packing of the layers, the crystal can be formed of mainly cristobalite or mainly tridymite [31]. When foreign elements enter the cristobalite, polytypism is observed. Polytypism is a partial change in the stacking of one of the crystallographic layers, in which an alternation of the cristobalite type and the tridymite type occurs. Polytypism manifests itself in the appearance of a broad “hump” at about 2θ = 20.7° at the base of the main cristobalite peak [31,34,76], which was observed in the investigated glass samples that were heat-treated at 550 °C for 72 and 96 h (Figure 1). Polytypism is typical of the incorporation of alkalis into the cristobalite structure. The intensity of the α-Cr_2_O_3_ peaks for the 72 and 96 h treatment durations barely changed, compared to the samples treated at 550 °C for a shorter time. Therefore, it is highly unlikely that chromium atoms were partially incorporated into the “tridymite” layers. A significant part of chromium is rejected from the glass network as well-formed crystals of eskolaite. It is more likely that cristobalite is not chemically stabilized by chromium ions; instead, a catalytic crystallization occurs in which Cr_2_O_3_ acts as a nucleating agent and stimulates the formation of cristobalite, with tridymite layers that are stabilized by alkali ions. The FTIR spectrum of the sample containing cristobalite (Figure 4b) resembled the cristobalite spectrum more closely than that of tridymite, indicating that the crystallized silica was mainly cristobalite.

Preliminary studies of these samples via DTA suggest that the chromium and silica exited predominantly from the low-viscosity sodium and boron-rich phase of phase-separated glass, and after 96 h of exposure, the formation of cristobalite was complete [45]. All the investigated glass samples showed a well-defined phase separation with an interconnected structure (Figure 2), indicating that the volume of the boron-rich phase was around 50% [77]. Therefore, the observed crystallization of cristobalite occurred in the glass volume, not on its surface, which is unusual for cristobalite. 

SEM images with spherulitic cristobalite inclusions can be used to roughly estimate the growth kinetics. According to the authors of [29], the radii of the largest crystals can be measured for all durations of heat treatment. When the crystal sizes are plotted with respect to the heat treatment time at a constant temperature (550 °C), a straight line could be fitted to the data to obtain the linear crystal growth rates. The position where the line crosses the time axis can be taken as the incubation time for nucleation. As the spherulitic inclusions were only observed for two heat treatment times at a constant temperature, the dependence only had two points on the curve, which does not make for a precise calculation; however, in this study, we still used this as an approximate evaluation of the cristobalite growth rate. The lower the heat treatment temperature, the lower the crystal growth rate, and the longer the incubation time. The crystal growth rate appeared to be ~0.17 μm/h, and the incubation time was around 50 h, which finding was compliant with the results reported by Moğulkoç et al. [29], who determined these values for several temperatures from 660 to 850 °C.

Previously, we investigated an NBS glass with a similar composition that also contained iron oxide instead of Cr_2_O_3_-6Na_2_O·22B_2_O_3_·70SiO_2_·2Fe_2_O_3_ (mol %), which was heat-treated at 550 °C for 144 h and at 700 °C for 2 h [40,78]. It had a phase-separated structure with interpenetrating phases, and a small broad magnetite peak (C 39–1346) was observed on its diffraction pattern at 2θ = 35.6°. This peak sharpened when the temperature of the heat treatment was increased to 700 °C. No peaks of cristobalite were observed for either treatment condition. However, with an increase in Fe_2_O_3_ concentration from 4 to 8 mol %, cristobalite was detected at 700 °C [40]. When comparing the chromium-containing and iron-containing NBS glass, it is evident that the addition of Cr_2_O_3_ caused the formation of cristobalite at lower concentrations and temperatures, which is unusual. Both of these glasses had the same amounts of sodium oxide and silica; therefore, it is suggested that they may have had a similar composition for the boron-rich phase. The composition in this phase for the glass labeled 6/70 and without additives can be determined using an immiscibility diagram [77]. The 6/70 composition lies on a tie-line connecting two parts of the immiscibility isotherm. The ends of the tie-line mark the compositions of the immiscibility phases. The composition of the boron-rich phase, according to the diagram, was as follows (mol %): SiO_2_—38%, B_2_O_3_—46%, Na_2_O—16%. Previous studies of the leaching behavior of iron-containing glass in 3 M HCl solution showed that the majority of the Fe_2_O_3_ is incorporated in the boron-rich phase, as there was only 0.01 mol % Fe_2_O_3_ left in a porous glass after a one-step acid leaching process [61,79]. Iron oxide was introduced into the glass batch by the substitution of B_2_O_3_. According to the authors of [61], the overall glass composition lies within the range of those compositions where Fe^3+^ has tetrahedral coordination. This implies that the Fe^3+^ ions form [Fe^+3^O_4_]^−^ tetrahedra that are incorporated into the silica glass network. The negative charge of these groupings is compensated for by sodium ions that form [Fe^+3^O_4_]^-^Na^+^ complexes [42,80]. As there was only 2 mol % of Fe_2_O_3_, after incorporating the amorphous fraction of iron ions into the silicon-oxygen network, and after partial magnetite formation, there were still plenty of sodium ions left to convert the boron from trigonal to tetrahedral coordination, making the glass network more rigid and not allowing structural changes to occur, thereby inhibiting cristobalite formation.

In addition to the Cr^3+^ state, chromium ions can exist in higher oxidation states as Cr^5+^ and Cr^6+^, forming [CrO_4_]_2-_ and [CrO_4_]_3-_ units, respectively, and playing the role of a network-former [3]. Cr^3+^ ions form [CrO_6_] units, acting as a modifier in the glass. A chromium ion in the Cr^5+^ state is a paramagnetic ion that has an electron configuration of 3d^1^ and an electron spin of S = l/2. Therefore, it can be detected easily via EPR. The Cr^6+^ ion is diamagnetic and has an electron configuration of 3d^0^; therefore, it cannot be detected via EPR [3]. The EPR results in this study (Figure 3) suggested the possibility of a Cr^5+^ oxidation state. However, the XPS data did not support that hypothesis, indicating that the chromium ions in the investigated glasses existed only in the form of octahedrally coordinated Cr^3+^ ions. Therefore, chromium acted as a modifier in this glass, probably needing 3NBOs and 3BOs to compensate for its coordination, which is analogous to aluminum [36]. It is known that when isothermally treating a glass that is prone to phase separation for an extended period of time, the duration that is needed for the phases of such a glass to reach their equilibrium compositions can be determined by measuring the glass transition temperature (*T*_g_) (by DTA or dilatometry). When the *T*_g_ values stop changing along with extending the exposure time of heat treatment, this indicates that the required duration is reached [77]. The subsequent heat treatment would lead to a coarsening of the liquation channels and changes in phase morphology, but the composition would cease to change. Preliminary studies of these samples via DTA [45] showed that the *T*_g_ values for 24 h and 48 h were almost the same (falling within the measurement error). At 48 h, coarsening of the liquation channels began to occur (Figure 2) as their diameter increased from ~28 nm (for 24 h) to ~52 nm. The subsequent heat treatments for 72 and 96 h caused structural changes to occur within the boron-rich phase: the bond configurations of the next-nearest boron neighbors began to alter (Figure 5b) and the number of BO_3 ring_ and BO_3 non-ring_ units increased, while the BO_4_(3Si, 1B), Q^4^ (3Si,1BO_4_), and Q^4^(3Si, 1BO_3_) units decreased, suggesting the detachment of the Si-O^-^ part from the BO_4_(3Si, 1B) unit. Simultaneously, the NBO fraction attached to the silica networks (Si-O^-^Na^+^) increased, compensating for the negative charge (Figure 6a), and the BO fraction decreased (Table 3). Then, Q^4^(4Si) groupings appeared. A certain portion of the chromium ions was rejected from the glass network in the form of α-Cr_2_O_3_, which has similar lattice parameters to cristobalite (α-Cr_2_O_3_: a = 4.958, c = 13.58; cristobalite: a = 4.964, c = 6.895). According to the authors of [81], the similarity among the lattice parameters between the target phase and the nucleating agent has a better catalytic effect on crystallization. Supposedly, the combined factors of the formation of Q^4^(4Si) units and the similarity of the lattice parameters of eskolaite and cristobalite caused the formation of cristobalite. 

## 5. Conclusions

The crystallization of cristobalite in sodium borosilicate glass with a composition (mol %) of 6Na_2_O·22B_2_O_3_·70SiO_2_·2Cr_2_O_3_, synthesized using conventional melting, was studied via XRD, SEM, EPR, FTIR-spectroscopy, XPS, and solid-state NMR, according to the thermal history (quenching, annealing, and heat treatment at 550 °C for 24–96 h and at 700 °C for 2 h). This glass has a phase-separated structure with interpenetrating phases for all the heat-treatment conditions. The only phase containing chromium crystallizing in this glass was eskolaite, α-Cr_2_O_3_. This stimulates the bulk crystallization of cristobalite at 550°C, which is an extremely low temperature for its appearance (lower than that for iron-containing glass of similar composition). The cristobalite crystallizes in the low-viscous boron-rich phase of the phase-separated glass, which determines the bulk character of the crystallization. Polytypism, which resulted in the incorporation of alkalis into the cristobalite structure, appeared in the form of a “hump” at about 2θ = 20.7°, at the base of the left side of the main cristobalite peak in the diffraction pattern of the samples that were heat-treated at 550 °C for more than 72 h. The crystal growth rate and the incubation time for the crystallization of cristobalite were roughly estimated to be ~0.17 μm/h and 50 h, respectively. From a practical point of view, it is not recommended to heat-treat chromium-containing NBS glass at 550 °C for more than 48 h, as the crystallization of cristobalite could compromise the mechanical strength of the material.

## Figures and Tables

**Table 2:** Binding energies and FWHMs in the core-level photoelectron spectra of O1s, B1s, Si2p, Na1s, and Cr2p for the annealed glass and those samples that were heat-treated at 550 °C for 24 and 96 h.

Glass Designation	Binding Energy (eV) (FWHM (eV))						
	O1s BO 1s	O1s NBO 1s	Na1s	B1s	Si2p	Cr2p	O1s MeO
Annealed	532.80 (1.82)	531.98 (1.90)	1072.17 (2.20)	193.30 (1.99)	103.42 (1.78)	576.50 (3.76)	530.05 (1.60)
550 °C, 24 h	532.78 (1.79)	531.89 (1.72)	1072.10 (2.16)	193.09 (1.97)	103.38 (1.83)	576.65 (3.93)	530.22 (1.69)
550 °C, 96 h	532.71 (1.81)	531.86 (1.78)	1071.89 (2.07)	192.99 (1.88)	103.31 (1.79)	576.51 (3.43)	530.28 (1.70)

**Table 3:** Percentage of structural units, by ^11^B NMR and ^29^Si NMR, for the studied samples.

Percentage, %	Quenched	Annealed	550 °C, 24 h	550 °C, 96 h
Boron units				
BO_3 ring_	20.33	32.91	32.34	35.46
BO_3 non-ring_	36.38	32.91	32.34	35.46
BO_4_(3Si, 1B)	38.22	34.17	35.32	29.08
BO_4_(4Si)	5.07	-	-	-
Silicon units				
Q^4^ (3Si,1BO_4_)	23	34	52	44
Q^4^(3Si, 1BO_3_)	73	66	44	29
Q^4^(4Si)	4	-	4	27

## Data Availability

Not applicable.
